# Myocardial T1 mapping in asymptomatic subjects: variations according to left ventricular segments and correlation with cardiovascular risk factors

**DOI:** 10.1186/1532-429X-18-S1-P119

**Published:** 2016-01-27

**Authors:** Moon Young Kim, Soo Jin Cho, Yeon Hyeon Choe, Hae Jin Kim, Sung Mok Kim, Sang-Chol Lee

**Affiliations:** 1 grid.414964.a0000000106405613Radiology, Samsung Medical Center, Seoul, Korea (the Republic of); 2 grid.414964.a0000000106405613Cardiology, Samsung Medical Center, Seoul, Korea (the Republic of)

## Background

To evaluate whether there is variation in precontrast and postcontrast myocardial T1 time (prT1 and poT1, respectively) and extracellular volume fraction (ECVF) according to left ventricular (LV) segments and to search for any correlation between them and known cardiovascular risk factors.

## Methods

This study included 198 asymptomatic subjects (180 men and 18 women, age 54.4 ± 6.12 years) who underwent cardiac MR imaging. Precontrast T1 mapping and postcontrast T1 mapping 15 minutes after 0.2 mmol gadobutrol injection were performed using shortened modified look-locker inversion recovery [ShMOLLI] sequence at 1.5T (Magnetom Avanto, Siemens). Short-axial cine MR imaging was performed with SSFP technique. T1 values and ECVFs were calculated in 16 AHA myocardial segments. Those values were compared among LV segments and correlated with presence of hypertension (n = 52), diabetes mellitus (DM, n = 15), or both (n = 17). ECVF was also correlated with LV mass.

## Results

The overall prT1 and poT1 values and ECVF were 1006 ± 291.5 ms, 454.2 ± 38.5 ms, and 0.24 ± 0.04, respectively. There was significant difference between apical segments and mid-basal segments in poT1 value and ECVF (p < 0.03) and between mid-septal segments and mid-lateral segments in T1 values and ECVF (p <0.04). ECVF showed reverse correlation with LV mass (p = 0.002). There was significantly lower poT1 value (449 ± 35.6 ms) and higher ECVF (0.24 ± 0.04) in subjects with hypertension compared with those (459 ± 43.3 ms and 0.23 ± 0.02) of subjects without hypertension (p < 0.05). Subjects with DM showed no difference in all T1 values from subjects without DM or hypertension, except poT1 values in mid-septal segments (447 ± 23.6 ms vs 459 ± 45.6 ms, p = 0.02). Subjects with both risk factors showed no difference in all T1 values from subjects without DM or hypertension, except prT1 value between apical septal and lateral segments (1007 ± 126 ms vs 999 ± 156 ms, p = 0.03).

## Conclusions

The septal wall showed higher prT1 value and ECVF but lower poT1 value than the lateral wall of mid- and basal levels. PoT1 value and ECVF are significantly affected by hypertension and LV mass.

